# Disease Specific to Chronic Lymphedema and Class III Obesity

**DOI:** 10.1155/2020/9234183

**Published:** 2020-04-16

**Authors:** Karen G. Figenshau, Matthew B. Lindquist

**Affiliations:** ^1^University of Missouri Kansas City School of Medicine, Kansas City 64108, USA; ^2^Department of Internal Medicine, University of Missouri Kansas City School of Medicine, Kansas City 64108, USA

## Abstract

Elephantiasis nostras verrucosa (ENV) is a unique, chronic condition found in patients with obesity and chronic secondary lymphedema. It develops due to chronic inflammation and recurrent infection, most commonly on gravity-dependent sites. Progressive tissue enlargement, deformity, and disability necessitate intervention. First, clinicians should explore the etiology of patients' secondary lymphedema, as this is paramount in determining treatment for ENV. The fundamental goal is alleviating lymphatic obstruction. Our literature review of available cases of ENV elucidates Class III obesity as a factor common to all available cases of ENV. As such, weight loss is a key component of treatment. Medical management and weight loss are most effective when combined with physiologic interventions such as compression garments and decongestive physiotherapy. If surgical intervention is required, one of the mainstays of patient management is a reductive approach, aimed at removing excess adipose and fibrotic tissue to improve lymphatic patency and flow. Optimal postoperative outcomes are achieved when patients also undergo physiologic procedures to bypass obstructions and connect functioning lymphatic vessels.

## 1. Introduction

Elephantiasis nostras verrucosa is a rare complication of chronic secondary lymphedema [1]. We will present a case report and review of the disease with a focus on obesity. Our literature review suggests that ENV may be a complication specific to chronic secondary lymphedema in the setting of class III obesity (BMI >/ = 40 kg/m^2^). We hope that our identification of commonalities among our case and available reports of ENV can help to elucidate a patient population at risk and help to guide management.

## 2. Case

A 45-year-old female with a history of type 2 diabetes mellitus, hypertension, obstructive sleep apnea, class III obesity (BMI 86.2 kg/m^2^), and chronic right lower extremity lymphedema presented to the hospital for recurrent right lower extremity soft tissue infections. She had been treated with amoxicillin and clavulanic acid 875 mg/125 mg twice over the previous six months with no improvement of pain, redness, and drainage. One month prior to admission, she was prescribed a 7-day course of oral doxycycline 100 mg twice daily with temporary improvement of symptoms but immediate return upon discontinuation. After failing another course of doxycycline with metronidazole, she was subsequently admitted with worsening cellulitis and fevers.

She denied any travel outside of the United States, and she lived in Missouri her whole life. She did report having a dog and cat at home. She did not recall any cat bites, but reported the dog occasionally licked her leg wounds.

During her admission, she was found to have *Streptococcus agalactiae* bacteremia. The infectious disease service was consulted and, due to nodularity of skin with possibility of chromoblastomycosis versus malignancy, a biopsy of her leg was obtained. Pathology returned consistent with elephantiasis nostras verrucosa. Bacterial cultures were obtained and grew methicillin-susceptible *Staphylococcus aureus* and *Morganella morganii*, though the samples were obtained 2 days following initiation of intravenous antimicrobial therapy. Fungal cultures grew *Candida parapsilosis*. At the time of her most recent admission, the patient had green-tinted exudates from the right lower extremity. Wound cultures grew *Pseudomonas* and *Enterococcus faecalis*. She was treated appropriately based on culture sensitivities with a prolonged antibiotic course via a peripherally inserted central catheter. At the time of discharge, arrangements were made for wound care and compression therapy.

## 3. Discussion

ENV is a rare skin condition due to chronic lymphedema that leads to progressive tissue enlargement, deformity, and disability without intervention.

### 3.1. Pathophysiology

Most commonly occurring on gravity-dependent areas, ENV presents with nonpitting edema, enlargement, lichenification, and superimposed hyperkeratotic papules and nodules with verrucous or cobblestone-like appearance [2]. Histologically, early stages of ENV show dilated lymph channels, loss of dermal architecture, and widened tissue spaces. Later stages show disrupted elastic fibers of the dermis. Eventually, lesions develop extensive fibrosis of the dermis, subcutaneous tissue, and lymph vessels [3].

In patients with obesity, fat accumulation obstructs lymphatic vessels, increases weight, tension, and intra-abdominal pressure, and further impairs lymphatic drainage. Prolonged lymphostasis leads to lymphedema development [4]. Leakage of protein-rich fluid into the interstitium induces fibroblast proliferation and blunts local immune responses. Uncontrolled fibroblast proliferation leads to fibrosis of the dermis and subcutaneous tissue [5]. Impaired local immune response creates an environment susceptible to lymphangitis. Recurrent inflammation, most commonly due to streptococcal infection, leads to further fibrosis and worsening lymphedema [6]. Cyclical inflammation leads to progressive deformity and disability without intervention.

## 4. Differential Diagnoses

The differential diagnoses for ENV include venous stasis dermatitis, lipedema, lipodermatosclerosis, pretibial myxedema, filariasis, and chromoblastomycosis [7].

## 5. Evaluation and Diagnosis

Diagnosis of ENV primarily depends on patient history, physical exam, and characteristic cutaneous lesions. Typically, ENV will begin on the dorsal foot and progress proximally. Lesions usually present as mild, persistent, pitting edema, later becoming nonpitting, hypertrophic, and progressively fibrotic [8]. History will likely include repeated episodes of soft tissue infection in the affected area [6].

All patients presenting with verrucous lesions should undergo skin biopsy to rule out malignancy. Additionally in these patients, TSH, fungal cultures, peripheral blood smear, and a CBC with differential to evaluate for eosinophilia should be collected [9].

Clinicians should also evaluate for the cause of secondary lymphedema, which, in addition to obesity, can include neoplasms, congestive heart failure, trauma, radiation, filarial infection, and hypothyroidism [7]. Identification of the underlying cause of lymphedema is paramount in determining treatment for ENV.

The fundamental goal of management is to alleviate the underlying cause of lymphatic obstruction [8]. Additionally, general management of lymphedema includes decongestive physiotherapy with compression garments and bandages and mechanical lymphatic drainage to aid in alleviating lymphostasis. Diuretics can further reduce edema. Systemic antibiotics may be required to control infection. Topical keratolytics can be used adjunctively to reduce hyperkeratotic plaques [8].

### 5.1. Obesity

We conducted searches in PubMed (using the search terms elephantiasis nostras verrucosa, lymphedema, and obesity) to gather available data regarding ENV and its historical management. Our literature review of ENV case reports elucidated Class III obesity as a common denominator in all cases [2–7,9–14]. As such, the mainstay of treatment in ENV involves wound care, compression therapy, and weight loss [8]. All patients should conceivably receive treatment of obesity as first-line therapy. Assuming the patient will have Class III obesity as has been demonstrated in each case of ENV we discovered in the literature, appropriate referral to a bariatric physician and possibly surgeon will be important for necessary weight loss. While surgical options exist, patients with Class III obesity often have comorbidities making them poor surgical candidates. Importantly, obesity complications such as diabetes mellitus and extensive fibrosis also impair wound healing. Fife et al. discovered that patients who fail to achieve significant weight reduction, or who are poorly adherent to daily compression therapy, have recurrence and worsening of lymphedema postoperatively [8].

### 5.2. Treatment

The two major surgical approaches for advanced lymphedema are reductive and physiologic. Reductive options include liposuction, lipectomy, and skin excision. Removal of excess adipose and fibrotic tissue allows for improved lymphatic patency and flow [15]. Modolin documented four patients with massive localized lymphedema who underwent limited total resection of affected areas including a 1 cm margin of grossly uninvolved tissue. Despite prolonged postoperative courses with minor complications such as partial skin dehiscence and lymphatic leakage, all patients showed improved function and lack of recurrence six months postoperatively [16]. Physiologic options include lymphovenous or lymphatic anastomosis and vascularized lymph node transfer. These options restore circulation by bypassing obstructions and connecting functioning vessels [15]. Carl determined that surgical management can be effective for all clinical stages of lymphedema with proper pre- and postoperative management. Combined reductive and physiologic approaches provide the best outcomes, in conjunction with continued weight loss and compression therapy [15].

## 6. Metabolic/Bariatric Surgery

With regard to the potential of metabolic/bariatric surgery (MBS) as treatment, we performed a literature search of ENV and MBS, which produced no case reports to date. As Fife et al. described, weight loss is a strong predictor of postoperative success after surgical intervention of lymphedema. As MBS aids much more robust weight loss compared to nonsurgical interventions, consideration should be made for MBS whether pre- or postoperative from lymphedema surgery or if treating conservatively.[17]. Optimal timing, of course, will need to be further delineated.

## 7. Conclusion

Our case prompted consideration of commonalities among patients with ENV. This report, accompanied by our review of the available literature, illustrates the importance of Class III obesity in the pathogenesis of elephantiasis nostras verrucosa. We hope that this report will help to elucidate a population at risk for ENV and to guide management in these patients.
